# Keeping prior anticoagulation treatment in the acute phase of ischaemic stroke: the REKOALA study

**DOI:** 10.1007/s00415-024-12204-8

**Published:** 2024-04-05

**Authors:** Ricardo Rigual, Jorge Rodríguez-Pardo, Manuel Lorenzo-Diéguez, Susana Fernández-Fernández, Gabriel Torres Iglesias, Clara Lastras, Gerardo Ruiz-Ares, María Alonso de Leciñana, Elena de Celis, Laura Casado-Fernández, Carlos Hervás, Elisa Alonso, Exuperio Díez-Tejedor, Blanca Fuentes

**Affiliations:** 1 Universidad Autónoma de Madrid, Madrid, Spain; 2 Department of Neurology and Stroke Centre, Hospital La Paz Institute for Health Research-IdiPAZ (La Paz University Hospital, Universidad Autónoma de Madrid), Madrid, Spain; 3 Department of Radiology, Hospital La Paz Institute for Health Research-IdiPAZ (La Paz University Hospital-Universidad Autónoma de Madrid), Madrid, Spain

**Keywords:** Cardioembolic stroke, Acute ischaemic stroke, Anticoagulant therapy, Continuing anticoagulant therapy, Stroke recurrence, Haemorrhagic risk

## Abstract

**Introduction:**

A consensus on the management of anticoagulated patients in the acute phase of ischaemic stroke has not yet been established. We aimed to evaluate clinical outcomes in such patients based on the continuation or discontinuation of anticoagulation.

**Methods:**

Retrospective study of patients with acute ischaemic stroke and cardioembolic source receiving anticoagulant therapy is done. Patients were classified based on the continuation or discontinuation of anticoagulation at admission. Clinical outcomes, haemorrhagic and ischaemic events were assessed. Multivariate logistic regression analysis, propensity score matching (PSM) analysis and a sub-analysis of patients with severe ischaemic stroke at admission (NIHSS score ≥ 15) were performed.

**Results:**

Anticoagulation was continued in 147 (78.8%) of 186 patients. Patients continuing anticoagulant had lower NIHSS (median 5 vs 18, *p* < 0.001). There were no differences in haemorrhagic or ischaemic events. In the multivariate analysis, good functional outcome at discharge was higher in the continuation group, OR (CI95%) 3.77 (1.2–11.2). PSM analysis adjusted for potential confounders such as NIHSS had higher rates of good functional outcomes at discharge (80% vs 36%, *p* = 0.004) and at 90 days (76% vs 44%, *p* = 0.042) in the continuation group. Patients with severe stroke in this group had lower 90-day mortality (34.6% vs 62.5%, *p* = 0.045) and higher rates of good clinical outcome at discharge (33.3% vs 8.3%, *p* = 0.032). No differences were observed in 90-day haemorrhagic or ischaemic events.

**Conclusion:**

Continuation of anticoagulation in patients with acute ischaemic stroke and cardioembolic source did not increase the risk of intracranial haemorrhage and may be associated with better functional outcomes.

## Introduction

Cardioembolic stroke represents approximately 20% of all ischaemic strokes, with atrial fibrillation representing the most frequent cause. It is usually associated with greater stroke severity, higher risk of ischaemic recurrence and haemorrhagic transformation and worse clinical outcomes [1].

Current international guidelines as the European Stroke Organisation and the American Stroke Association/American Heart Association recommend oral anticoagulant (OAC) therapy with vitamin K antagonists (VKA) or direct oral anticoagulants (DOACs), such as direct thrombin (Dabigatran) and activated factor X inhibitors (Apixaban, Rivaroxaban and Edoxaban), for stroke prevention in patients with atrial fibrillation or other major cardioembolic sources. However, even with this preventive treatment, such patients continue to be at risk for ischaemic stroke, with an absolute risk of approximately 1 to 2.4% per year [2]. Stroke risk is even greater in patients with a history of ischaemic stroke despite anticoagulant therapy, reaching up to 4.7% cumulative incidence per year of stroke recurrence [3] and 17% cumulative incidence at 3-year follow-up [4]

In patients with recent cardioembolic stroke not previously on anticoagulant therapy, the timing for its initiation remains unclear. In daily clinical practice, stroke physicians adjust the decision to start anticoagulation based on stroke volume in initial neuroimaging and stroke severity as measured by the National Institutes of Health Stroke Scale (NIHSS). Thus, the timing ranges from 3 days in the case of small or mild cerebral infarcts to more than 12–14 days in patients with extensive infarcts, haemorrhagic transformation or severe neurological deficits [5]. Although the main reason for delaying initiation of anticoagulant therapy is the risk of ICH, an individual patient data pooled analysis of seven observational studies has reported similar ICH rates between patients with early and late initiation of DOACs, suggesting that an early start could be reasonable to prevent ischaemic recurrence [6]. Similarly, in a recent open label trial of patients randomised to early or later anticoagulation, the composite incidence of recurrent ischaemic stroke, systemic embolism, major extracranial bleeding, symptomatic intracranial haemorrhage or vascular death at 30 days ranged from − 2.8 to + 0.5% in early anticoagulated patients compared with those in whom DOACs were implemented later [7]. Other ongoing clinical trials comparing early to late OAC in cardioembolic stroke will help us answer this question in the years to come [8, 9].

In patients previously undergoing anticoagulant therapy at the time of stroke onset, the initial management of antithrombotic therapy is even more unclear as there are no specific recommendations in the international guidelines. Both strategies (continuation or discontinuation and delayed restart) are accepted, and the choice of treatment varies according to the different protocols of each Stroke Unit or at the discretion of the attending stroke neurologist. Although the rationale for continuing anticoagulation is to prevent early ischaemic recurrence, it may also increase the risk of haemorrhagic transformation.

A post hoc analysis of the Preventive Antibiotics in Stroke Study (PASS) in the Netherlands included 192 patients with acute ischaemic stroke of cardioembolic origin on anticoagulant treatment at the time of admission [10]. This study compared the clinical outcomes of patients in whom anticoagulation was continued with those in whom it was discontinued. An adjusted sensitivity analysis showed a greater probability of good clinical outcomes in patients in whom anticoagulant therapy was maintained, with 3% ischaemic recurrence at 90 days, compared to 11% in the group in which anticoagulants were discontinued; there were no cerebral haemorrhages in either group.

Considering the high risk of recurrent ischaemia that may exceed the haemorrhagic risk in the context of a major cardioembolic source, continuing prior anticoagulant therapy could improve patient outcomes. The aim of this study was to compare the safety and efficacy of the continuation of anticoagulant therapy with its discontinuation in the acute phase of ischaemic stroke in patients with a major cardioembolic source.

## Patients and methods

The REKOALA study (Retrospective Evaluation of Keeping Prior Oral or Parenteral Anticoagulation in the Acute Phase of ischaemic Stroke and Major Cardioembolic Source) is a retrospective observational cohort study of adult patients with acute ischaemic stroke with a major cardioembolic source undergoing anticoagulant therapy admitted to a Stroke Centre between January 2014 and December 2021. Patients who had received intravenous thrombolysis requiring the interruption of anticoagulation, patients on VKA treatment, with an International Normalized Ratio (INR) of ≤ 1.7 or with haemorrhagic transformation in the initial cranial-CT were excluded. Patients on DOAC therapy were included if the patient had received the last dose within 24 h prior to stroke onset. Discontinuation was defined as the interruption of anticoagulant therapy for at least 24 h after the stroke.

Patients were classified into two groups based on whether anticoagulant therapy was continued or discontinued (continuation and discontinuation) at the discretion of the attending stroke neurologist. Each group was then assessed for safety and efficacy outcomes. Primary safety outcomes were measured in terms of mortality and symptomatic intracranial haemorrhage at 90 days. Primary efficacy outcomes were good functional outcome (defined as a modified Rankin scale score of 0–2 points) and ischaemic stroke recurrence at 90 days. Secondary end points were haemorrhagic transformation in control neuroimaging during hospitalisation (following the classification by Fiorelli et al. [11]), symptomatic intracranial haemorrhage (defined as a worsening of the neurological status attributable to haemorrhagic transformation of the stroke or new neurological symptoms caused by ICH), ischaemic stroke recurrence, good functional outcome and mortality at discharge and systemic embolism or major extracranial bleeding at discharge and 90-day follow-up. Control neuroimaging was performed as per standard care protocol in all patients. All neuroimaging data were examined by a neuroradiologist blinded to the anticoagulant therapy received.

Statistical analysis was performed using SPSS 29.0 for Windows (SPSS Inc., Chicago, IL). First, we carried out a descriptive analysis of the total cohort. Categorical variables were presented as proportions and continuous variables as the mean ± standard deviation (SD) or median with interquartile ranges (IQR). Baseline and demographic characteristics were then analysed and compared between the two treatment groups. The comparison of categorical variables used × 2 or Fisher’s exact test where appropriate, whilst that of quantitative variables applied the ANOVA test and were adjusted post hoc with a Bonferroni correction. We performed intention-to-treat as well as multivariate logistic regression analyses, adjusted by statistically significant (*p* < 0.05) variables between groups in a univariate analysis. In addition, we built a propensity score-matched cohort using a 1:1 ratio and a 0.2 tolerance for variables that were significantly different between groups. Outcomes of the treatment groups were compared in the score-matched cohort. Given the retrospective nature of the study, we also performed a sub-analysis of patients with severe ischaemic stroke, defined as NIHSS ≥ 15 at admission.

The study was approved by the local ethics committee and by the Spanish Agency of Medicines and Medical Devices. Data supporting the findings of this study are available from the corresponding author upon request.

## Results

186 patients were included in the study. Anticoagulation was continued in 147 (78.8%) and discontinued in 39 (21.2%) patients. Table 1 shows the epidemiological, clinical and radiological characteristics of both cohorts. Patients in whom anticoagulation was continued had lower NIHSS scores at admission (median [IQR], 5 [2–11] vs 18 [10–23], *p* < 0.001), higher initial Alberta Stroke Program Early CT Scores (ASPECTS) (median [IQR], 10 [8–10] vs 8 [7–10], *p* = 0.001), less frequent large vessel occlusion (LVO) (49.5% vs 86.1%, *p* = 0.001) and lower rates of mechanical thrombectomy (15.6% vs 64.1%, *p* < 0.001). Patients who discontinued anticoagulant therapy at admission received anticoagulation at discharge less frequently (98.6% vs 83.9%, *p* = 0.002).

**Table 1:** Epidemiological, clinical, and radiological characteristics

Demographic data	Total cohort *N* = 186 (100)	Continuation AT *N* = 147 (78.8)	Discontinuation AT *N* = 39 (21.2)	*P*
Sex female, *n* (%)	76(40.9)	57 (38.8)	19 (48.7)	0.277
Age: years, mean ± SD	78.7 ± 7.9	78.7 ± 8.3	78.0 ± 7.5	0.481
Hypertension, *n* (%)	158 (84.9)	124 (84.4)	34 (87.2)	0.800
Diabetes Mellitus, *n* (%)	59 (31.7)	46 (31.3)	13 (33.3)	0.848
Dyslipidaemia, *n* (%)	121 (65.1)	97 (66)	24 (61.5)	0.706
Previous TIA or IS, *n* (%)	65 (34.9)	52 (35.4)	13 (33.3)	0.853
Previous cerebral haemorrhage, *n* (%)	2(1.1)	2 (1.4)	0 (0)	0.46
Active cancer, *n* (%)	21 (11.3)	19 (12.9)	2 (5.1)	0.256
Cardioembolic source, *n* (%) AF Prosthetic Valve	182 (97.8) 23 (12.4)	144 (98) 15 (10.2)	38 (97.4) 8 (20.5)	0.849 0.101
Anticoagulant Treatment, *n* (%) VKA Dabigatran Apixaban Rivaroxaban Edoxaban Heparin	106 (57) 11 (5.9) 25 (13.4) 23 (12.4) 8 (4.3) 13 (7)	87 (59.2) 7 (4.8) 18 (12.2) 17 (11.6) 8 (5.4) 10 (6.8)	19 (48.7) 4 (10.3) 7 (17.9) 6 (15.4) 0 (0) 3 (7.7)	0.365
mRS at admission; median (IQR)	0 (0–1)	0 (0–1)	0 (0–1)	0.147
NIHSS score, median (IQR)	6 (3–16)	5 (2–11)	18 (10–23)	** < 0.001**
Wake-up stroke, *n* (%)	65 (34.8)	52 (35.4)	14 (35.9)	0.596
ASPECTS, median (IQR)	10 (8–10)	10 (8–10)	8 (7–10)	**0.001**
Leukoaraiosis	124 (66.7)	100 (68)	24 (61.5)	0.451
Ipsilateral ICA Stenosis > 50%	7(3.8)	4(2.7)	3(7.7)	0.341
Intracranial LVO, *n* (%)	81 (59.1)	50 (49.5)	31 (86.1)	** < 0.001**
Mechanical thrombectomy, *n* (%)	48 (25.8)	23 (15.6)	25 (64.1)	** < 0.001**
Lacunar index stroke	11 (6.1)	10 (6.9)	1 (2.7)	0.465
Anticoagulation at discharge, *n* (%)	165 (95.9)	139 (98.6)	26 (83.9)	**0.002**

*AT* anticoagulant treatment, *TIA* transient ischemic attack, *IS* ischemic stroke, *AF* atrial fibrillation, *mRS* modified Rankin scale, *NIHSS* National Institutes of Health Stroke Scale, *ASPECTS* Alberta Stroke Program Early CT Score, *ICA* internal carotid artery, *LVO* large vessel occlusion

Two patients (1%) did not complete the 90-day follow-up. Primary and secondary outcomes are listed in Table 2. In the primary outcome analysis, higher rates of favourable functional outcome (70.3% vs 35.9%, *p* < 0.001) and lower 90-day mortality (10.3% vs 43.6%, *p* < 0.001) were observed in the anticoagulant continuation group compared to the discontinuation group. ICH incidence rates at 90 days follow-up were 1.4% in the continuation group and 7.7% in the discontinuation group (*p* = 0.065). Ischaemic stroke recurrence at 90 days was 2.8% and 7.7% in the continuation and discontinuation groups, respectively (*p* = 0.163). In secondary outcomes, anticoagulant continuation was associated with a lower rate of haemorrhagic transformation in control neuroimaging (13.2% vs 29.7%, *p* = 0.026), lower mortality (4.1% vs 20.5%, *p* = 0.002), higher rates of good functional outcome at discharge (66.7% vs 28.2%, *p* = 0.001) and lower rates of systemic embolism at 90 days (0% vs 10.3%, *p* = 0.002).

**Table 2:** Primary and secondary outcomes at discharge and at 90 days

Primary outcomes	Total cohort N = 186 (100)	Continuation AT N = 147 (78.8)	Discontinuation AT N = 39 (21.2)	P
Mortality at 90 days, *n* (%)	32 (17.4)	15 (10.3)	17 (43.6)	** < 0.001**
Good functional outcome (mRS 0–2) at 90 days, *n* (%)	116 (63)	102 (70.3)	14 (35.9)	** < 0.001**
Symptomatic intracranial haemorrhage at 90 days, *n* (%)	5 (2.8)	2 (1.4)	3(7.7)	0.065
Recurrent ischaemic stroke at 90 days, *n* (%)	7 (3.8)	4 (2.8)	3 (7.7)	0.163

Secondary outcomes	Total cohort *N* = 186 (100)	Continuation AT *N* = 147 (78.8)	Discontinuation AT *N* = 39 (21.2)	*P*
Haemorrhagic transformation in control neuroimaging, *n* (%)	27 (17.1)	16 (13.2)	11 (29.7)	**0.026**
Symptomatic intracranial haemorrhage at discharge, *n* (%)	3 (1.6)	1(0.7)	2 (5.1)	0.112
Recurrent ischaemic stroke at discharge, *n* (%)	4 (3.2)	3 (2)	1 (2.6)	0.631
In-hospital mortality, *n* (%)	14 (7.5)	6 (4.1)	8 (20.5)	**0.002**
Good functional outcome (mRS 0–2) at discharge, *n* (%)	109 (58.6)	98 (66.7)	11 (28.2)	**0.001**
Major extracranial bleeding at discharge, *n* (%)	1 (0.5)	1 (0.7)	0 (0)	0.606
Systemic embolism at discharge, *n* (%)	2 (1.1)	0 (0)	2 (5.1)	0.055
Major extracranial bleeding at 90 days, n (%)	4 (2.2)	3 (2.1)	1(2.6)	0.618
Systemic embolism at 90 days, *n* (%)	4 (2.2)	0 (0)	4 (10.3)	**0.002**

*AT* anticoagulant treatment, *mRS* modified Rankin scale

In the multivariate analysis, outcomes at discharge were adjusted by NIHSS scores at admission, ASPECTS, mechanical thrombectomy and intracranial LVO. Outcomes at 90 days were adjusted by NIHSS at admission, ASPECTS, mechanical thrombectomy, intracranial LVO and anticoagulant therapy at discharge. As may be observed in Table 3, continuing anticoagulation was independently associated with higher odds of good functional outcome at discharge (OR 3.77 (95% CI 1.2–11.2), with no significant differences in other outcomes.

**Table 3:** Adjusted outcomes (continuation vs discontinuation group)

Adjusted Outcomes: Continuation vs discontinuation group	OR CI (95%)	p
Good functional outcome (mRS 0–2) at 90 days^+^	1.54 (0.52–4.51)	0.430
Mortality at 90 days^**+**^	0.64 (0.18–2.28)	0.493
Good functional outcome (mRS 0–2) at discharge*	3.48 (1.2–10)	**0.021**
In-hospital mortality*	0.19 (0.03–1-01)	0.053
Haemorrhagic transformation in neuroimaging*	0.36 (0.11–1.16)	0.088
Systemic embolism at 90 days^**+**^	0 (0−.)	0.997

*mRS* modified Rankin scale

*Adjusted by NIHSS at admission, ASPECTS at admission, Mechanical thrombectomy, Intracranial LVO

**+ **Adjusted by NIHSS at admission, ASPECTS at admission, Mechanical thrombectomy, Intracranial LVO and anticoagulation at discharge

For direct comparison between the continuation and discontinuation groups, propensity score matching was performed including NIHSS scores and ASPECTS at admission, intracranial LVO, mechanical thrombectomy and anticoagulant therapy at discharge. Twenty-five patients from each group were matched. Table 4 illustrates the characteristics of the sample, without differences in epidemiological, clinical or radiological variables. The continuation of anticoagulant therapy was associated with a higher rate of good functional outcome at discharge (80% vs 36%, *p* = 0.004) and at 90 days (76% vs 44%, *p* = 0.042).

**Table 4:** Outcomes propensity score matching (adjusted by NIHSS, ASPECTS, MT, LVO, anticoagulant at discharge)

	Total PSM cohort (50)	Continuation *N* = 25	Discontinuation *N* = 25	*p*
Sex female, *n* (%)	24 (48)	12 (48)	12 (48)	1
Age: years, mean ± SD	77.8 (9.1)	78.1 (10.2)	77.5 (8.1)	0.496
Hypertension, *n* (%)	41 (82)	19 (76)	22 (88)	0.463
Diabetes mellitus, *n* (%)	12 (24)	4 (16)	8 (32)	0.321
Dyslipidaemia, *n* (%)	28 (56)	14 (56)	14 (56)	1
Previous TIA or IS, *n* (%)	19 (38)	12 (48)	7 (28)	0.244
Previous cerebral haemorrhage, *n* (%)	0 (0)	0 (0)	0 (0)	–
Active cancer, *n* (%)	5 (10)	4 (16)	1(4)	0.349
Cardioembolic source, *n* (%) AF Prosthetic valve	48 (96) 9 (18)	23 (92) 4 (16)	25 (100) 5 (20)	0.49
Anticoagulant treatment, *n* (%) VKA Dabigatran Apixaban Rivaroxaban Edoxaban Heparin	29 (58) 3 (6) 6 (12) 7 (14) 2 (4) 3 (6)	17 (68) 0 (0) 1 (4) 4 (16) 2 (8) 1 (4)	12 (48) 3 (12) 5 (20) 3 (12) (0) 2 (8)	0.109
mRS at admission; median (IQR)	0 (0–1)	0 (0–1)	0 (0–1)	0.626
NIHSS score, median (IQR)	12 (6–21)	10 (6–18)	15 (9–22)	0.137
Wake-up stroke, *n* (%)	15 (30)	8 (32)	7 (28)	0.587
ASPECTS, median (IQR)	9 (8–10)	10 (8–10)	8 (8–10)	0.354
Leukoaraiosis	31 (62)	14 (56)	17 (68)	0.561
ICA stenosis > 50%	2 (4)	0 (0)	2 (8)	0.380
Intracranial LVO, *n* (%)	41 (82)	20 (80)	21 (84)	1
Mechanical thrombectomy, *n* (%)	32 (64)	15 (60)	17 (68)	0.769
Lacunar index stroke	1 (2.1)	0 (0)	1 (4.3)	0.479
Anticoagulation at discharge, *n* (%)	49 (98)	25 (100)	24 (96)	1
Haemorrhagic transformation in neuroimaging	8 (17.8)	3 (14.3)	5(20.8)	0.705
Symptomatic intracranial haemorrhage	1 (2)	1 (4)	0 (0)	0.50
Recurrent ischaemic stroke	1 (2)	0 (0)	1 (4)	0.50
Major extracranial bleeding	0 (0)	0 (0)	0(0)	-
Systemic embolism	1 (2)	0 (0)	1(4)	0.50
Mortality at discharge	3 (6)	1 (4)	2 (8)	0.55
Good functional outcome (mRS 0–2) at discharge	29 (58)	20 (80)	9 (36)	**0.004**
Symptomatic intracranial haemorrhage at 90 days	2 (4.1)	1 (4.2)	1 (4)	0.745
Recurrent ischaemic stroke at 90 days	2 (4.1)	0 (0)	2 (8)	0.255
Major extracranial bleeding at 90 days	1 (2)	1 (4.2)	0 (0)	0.357
Systemic embolism at 90 days	3 (6.1)	0 (0)	3 (12)	0.125
Mortality at 90 days	12 (24)	4 (16)	8 (32)	0.321
Good functional outcome (mRS 0–2) at 90 days	30 (60)	19 (76)	11 (44)	**0.042**

*TIA* transient ischaemic attack, *IS* ischaemic stroke, *AF* atrial fibrillation, *mRS* modified Rankin scale, *NIHSS* National Institutes of Health Stroke Scale, *ASPECTS* Alberta Stroke Program Early CT Score, *ICA* internal carotid artery, *LVO* large vessel occlusion

In the sub-analysis of patients with severe ischaemic stroke (NIHSS score ≥ 15), we included 51 patients, 27 (52.6%) from the continuation group. In this analysis, we observed a significantly lower 90-day mortality rate (34.6% vs 62.5%, *p* = 0.045) and higher rate of good clinical outcome at discharge (33.3% vs 8.3%, *p* = 0.032) in the continuation group. Non-significant rates of lower intracranial haemorrhage and recurrent ischaemic stroke were observed in the continuation group at 90 days of follow-up (see Table 5).

**Table 5:** Primary and secondary outcomes at discharge and at 90 days in patients with NIHSS ≥ 15

	Total cohort *N* = 51 (100)	Continuation AT *N* = 27(52.9)	Discontinuation AT *N* = 24(47.1)	*P*
NIHHS, median (IQR)	21(18–23)	19 (17–22)	21.5 (18–24)	0.074
Primary outcomes				
Mortality at 90 days, *n* (%)	24 (48)	9 (34.6)	15 (62.5)	**0.045**
Good functional outcome (mRS 0–2) at 90 days, *n* (%)	11 (22.0)	7 (26.9)	4 (16.7)	0.501
Symptomatic intracranial haemorrhage at 90 days, *n* (%)	4 (8)	1 (3.8)	3 (12.5)	0.295
Recurrent ischaemic stroke at 90 days, *n* (%)	3 (6)	0 (0)	3 (12.5)	0.103

Secondary outcomes	Total cohort *N* = 51 (100)	Continuation AT *N* = 27(52.9)	Discontinuation AT *N* = 24(47.1)	*P*
Haemorrhagic transformation in control neuroimaging, *n* (%)	14 (30.4)	5 (20.8)	9 (40.9)	0.124
Symptomatic intracranial haemorrhage at discharge, *n* (%)	3 (5.9)	1 (3.7)	2 (8.3)	0.595
Recurrent ischaemic stroke at discharge, *n* (%)	1 (2)	0 (0)	1 (4.2)	0.471
In-hospital mortality, *n* (%)	11 (21.6)	4 (14.8)	7 (29.2)	0.184
Good functional outcome (mRS 0–2) at discharge, *n* (%)	11 (21.6)	9 (33.3)	2 (8.3)	**0.032**
Major extracranial bleeding at discharge, *n* (%)	1 (2)	1 (3.7)	0 (0)	0.529
Systemic embolism at discharge, *n* (%)	0 (0)	0 (0)	1 (4.2)	0.471
Major extracranial bleeding at 90 days, *n* (%)	2 (4)	1 (3.8)	1 (4.2)	0.735
Systemic embolism at 90 days, *n* (%)	3 (6)	0 (0)	3 (12.5)	0.103

*AT* anticoagulant treatment, *mRS* modified Rankin scale

## Discussion

Acute ischaemic stroke patients undergoing anticoagulation therapy at the time of admission represent a population with special characteristics. Intravenous thrombolysis is usually contraindicated in such cases, leaving mechanical thrombectomy as the only reperfusion treatment option in the case of large vessel occlusion. Furthermore, there is a possibility of an even higher risk of recurrence in cases of mechanical valve disease, recent myocardial infarction or intracardiac thrombus, making management of the acute phase of stroke in these patients a challenge. Doubts regarding the management of such patients are reflected in a British survey in which 95% of stroke physicians expressed uncertainty about the timing of anticoagulant therapy in cases of recent cardioembolic stroke [12]. The post hoc analysis of the PASS study, the only example in prior literature assessing the continuation of anticoagulation in the acute phase of cardioembolic stroke [10], suggested that continuation could improve functional outcomes. Our study is the first to suggest that the continuation of anticoagulant therapy in such patients may improve functional outcome without safety concerns irrespective of stroke severity measured by NIHSS or ASPECTS at admission.

In our study, we found a higher primary mortality rate compared to the literature, observing 17.4% mortality at 90 days, greater than that reported by a recent meta-analysis that found 12.5% 90-day mortality [13]. The data for our series starts in 2014, when mechanical thrombectomy was not yet widely established and there were fewer patients on DOAC, which could partially explain the higher mortality rate [14]. Stroke recurrence rate at 90 days was 3.8%, which is consistent with recent prospective studies and clinical trials: 3.1–4.5% in the Timing of Oral Anticoagulant Therapy in Acute Ischemic Stroke With Atrial Fibrillation (TIMING) registry [15], 1.9–3.1% in the Early Versus Late Initiation of Direct Oral Anticoagulants in Post-ischaemic Stroke Patients with Atrial Fibrillation (ELAN) trial [7], and 1.9–3.9% in the combined data of two prospective registries in Japan [16]. We found a 90-day ICH rate of 2.8%, especially marked in the discontinuation group, which is also higher than those reported by the studies previously mentioned (0–0.6%). Another prospective observational multicentre study, the Early Recurrence and Major Bleeding in Patients with Acute Ischemic Stroke and Atrial Fibrillation Treated with Non–Vitamin‐K Oral Anticoagulants (RAF‐NOACs) Study [17], reported a 90-day rate of 1.6% for symptomatic haemorrhagic transformation, similar than 1.4% found in the anticoagulant continuation group in our study. However, these studies were designed to compare early and delayed anticoagulation solely with DOACs, restricted to patients with atrial fibrillation and anticoagulated patients at stroke index represented a low percentage of the sample (21% in the Japanese cohort study [16], 27% in the TIMING registry [15] and was directly stated as an exclusion criterion in the ELAN trial [7]), so comparisons should be made with caution. Also, in our sample, VKA was used in 57% of patients at admission and it was continued in 37% at discharge. The Early Recurrence and Cerebral Bleeding in Patients with Acute Ischemic Stroke and Atrial Fibrillation (RAF) study that included only patients on VKA, observed a higher rate of intracranial haemorrhage (3.6%) than that found in our analysis [18].

Haemorrhagic transformation or symptomatic intracranial haemorrhage is the primary complication related to anticoagulant therapy in the acute phase of ischaemic stroke. It may be produced by the blood–brain barrier disruption following recent parenchymal ischaemic damage [19]. There is also a higher risk of major extracranial bleeding (such as gastrointestinal or urinary haemorrhage) that can occur more easily in critically ill patients. Several studies suggest a direct relationship between haemorrhagic risk and stroke severity, stroke size in neuroimaging and history of anticoagulant treatment [20], particularly in those receiving thrombolytic or endovascular therapy [21].

However, our study suggests that the maintenance of anticoagulant therapy does not entail a higher haemorrhagic risk compared to anticoagulation interruption. This fact is of particular interest in cases where the embolic risk is higher, such as in mechanical valves or intracardiac thrombus. Our results may support the stroke neurologist in deciding whether to continue anticoagulation in such patients, even in those with high NIHSS scores. We did not observe a higher risk of haemorrhagic complications in the continuation group. Despite the small sample size, we also found significantly lower mortality and non-significant rates of lower intracranial haemorrhage in patients with NIHSS ≥ 15 who continued anticoagulant therapy. One of the possible explanations for these findings could be avoiding the transition from non-anticoagulation to anticoagulation, when the risk of intracranial bleeding is expected to be higher.

Another interesting finding of our study was a higher rate of anticoagulation at discharge in patients who continued on anticoagulants. One prospective study compared the clinical outcomes of stroke patients based on whether they received anticoagulation at discharge, patients not anticoagulated at discharge had a 1.6-fold increase in the risk of death or dependency at 12 months and a 2.5-fold increase in the risk of stroke [22]. Regardless of stroke severity, long-term anticoagulation is usually recommended for secondary prevention of ischaemic stroke. The continuation of anticoagulant therapy at admission could help achieve this objective, avoiding a delayed restart and reducing the time outside therapeutic range.

Our study has several limitations. First, it is a retrospective observational study of patients from a single centre. Of the 189 patients included only 39 discontinued anticoagulant treatment, which limits the power of the study. Continuation of anticoagulant therapy was part of routine clinical practice at the study site, which could imply a selection bias. In our series, patients who discontinued anticoagulant therapy usually had greater stroke severity at admission, which in turn may have influenced the decision to interrupt anticoagulation in those with worse expected outcomes. The heterogeneity between groups was also found in initial ASPECTS, mechanical thrombectomy and anticoagulation rates at discharge. To reduce baseline differences and homogenise comparison groups, we used two statistical methods (multivariate analysis and propensity score matching) and performed a sub-analysis in patients with similarly high stroke severity. Nonetheless, given the confounding factors in the sample, these results should be interpreted with caution.

A number of questions remain unsolved, such the NIHSS score cutoff for continuing anticoagulant therapy, whether there are any differences regarding the type of treatment used (DOACs or VKAs), or the INR value from which the continuation of VKA treatment could be safer. Nevertheless, the results obtained allow us to continue researching in this direction. At this time, an ongoing prospective multicentre study comparing the clinical outcomes of patients based on the maintenance or discontinuation of anticoagulant therapy during the acute phase of cardioembolic stroke (Keeping Oral or Parenteral Anticoagulation in the Acute Phase of Cardioembolic Ischaemic Stroke, KOALA-IS, NCT05486351) will provide valuable insights to the management and decision-making strategies for this type of patients.

In conclusion, the continuation of anticoagulant therapy did not increase the risk of intracranial haemorrhage and may be associated with better functional outcomes in patients with acute ischaemic stroke with a major cardioembolic source, regardless of stroke severity at admission. Specifically designed prospective studies will be necessary to determine the optimal management of anticoagulation in these patients.

## Data Availability

The data that support the findings of this study are available from the corresponding author upon reasonable request.
